# Evaluation of Cardiac and Valvular Function after Arterial Switch Operation: A Midterm Follow-Up

**Published:** 2013-09-01

**Authors:** Hamid Amoozgar, Shirvan Salaminia, Ahmad Ali Amirghofran, Sirous Cheriki, Mohammad Borzoee, Gholamhossein Ajami, Farah Peiravian

**Affiliations:** 1 Cardiovascular Research Center, Shiraz University of Medical Sciences, Shiraz, IR Iran; 2 Division of Pediatric Cardiology, Department of Pediatrics, Shiraz University of Medical Sciences, Shiraz, IR Iran; 3 Department of Cardiac Surgery, Shiraz University of Medical Sciences, Shiraz, IR Iran; 4 Department of Pediatric, Islamic Azad University, Kazerun Branch, Kazerun, IR Iran

**Keywords:** Transposition of Great Vessels, Switch, Surgical Procedures, Follow-Up Studies, Echocardiography, Heart Function Tests

## Abstract

**Objectives:**

Transposition of Great Arteries (TGA) is a serious congenital heart disease and anatomic correction in the first few weeks of life has revealed good outcomes nowadays. In this study, we aimed to evaluate the myocardial and valvular function at midterm postoperative follow-up.

**Patients and Methods:**

In this study, thirty-three patients with TGA and Arterial Switch Operation (ASO) were evaluated by 2-dimensional, M-mode, Doppler, and pulsed Tissue Doppler. These patients were compared with 33 healthy children of the same age and gender as the normal control group. Student’s t-test and Pearson correlation were used to analyze the data. Besides, P<0.05 was considered as statistically significant.

**Results:**

The mean follow up time was 40.9±5.6 months. Among the 33 patients with ASO, 6% had mild pulmonary stenosis, while 3% had mild pulmonary insufficiency. Aortic stenosis and aortic insufficiency of trivial to mild degree was seen in 12% and 12% of the patients, respectively. The patients’ systolic velocity of tricuspid (S), early diastolic velocity of tricuspid (Ea), and late velocity of tricuspid valve (Aa) were significantly different from those of the controls (P<0.001). Also, pulmonary annulus diameter was significantly dilated in the patients compared to the controls (1.67±0.41 vs. 1.29±0.28, P≤0.001). Besides, aortic annulus diameter (1.56±0.42 vs. 1.24±0.21, P=0.001) and also aortic sinus diameter (2.06±0.41 vs. 1.44±0.34, P=0.002) were significantly dilated, while sinutuboar junction diameter (1.65±0.5 vs. 1.28±0.29, P=0.094) was not dilated. Left ventricular function was in the normal range.

**Conclusions:**

This study showed good left ventricular function, but some abnormalities in lateral tricuspid tissue Doppler velocities. Neoaortic and pulmonary diameters were significantly dilated, while aortic and pulmonary insufficiencies were clinically insignificant in most of the patients. Long-term follow-up is necessary in these patients.

## 1. Background

Transposition of the Great Arteries (TGA) is one of the most common congenital heart diseases, accounting for 5% to 7% of all congenital cardiac lesions resulting in cyanosis. In the past three decades, the Arterial Switch Operation (ASO) has become the preferred surgical procedure for TGA. After the first successful ASO by Jatene in 1975 and its modification by Lecompte et al., a considerable number of series have been reported (1).

A limited number of long-term studies revealed an excellent outcome and freedom from reoperation in the patients undergoing ASO (1, 2).However, in the ASO, the coronary arteries are translocated, the pulmonary valve becomes the systemic outflow valve, and the pulmonary arteries may be distorted because of the atypical relation between the great vessels. Also, concerns exist regarding the fate of coronary arteries (3), the function of neo-aortic valve (2, 3) , and the development of pulmonary artery stenosis (2). However, during the past 10 years, some clues have been gathered over the long and midterm problem of neonatal ASO, especially about the problem related to the neo-aortic valve (4), neo-aortic root enlargement, and the patency as well as function of the reimplanted coronary arteries (5, 6).

The present study aims to assess the midterm outcome of valvular function and evaluate the systolic and diastolic function of right and left ventricle in the patients undergoing ASO by pulse tissue Doppler in addition to 2-dimensional Doppler study.

## 2. Materials and Methods

### 2.1. Patient Population

The present study was conducted on 33 children who had been operated by a single surgeon in two hospitals (Shahid Faghihi and Dena hospitals, Shiraz, IR Iran) between January 2005 and December 2011. Written consents were obtained from the parents and the study was approved by the Ethics Committee of Shiraz University of Medical Sciences, Shiraz, IR Iran.

### 2.2. Control Group

The control group included 33 healthy children who referred to the cardiac clinic for cardiac evaluation with the same age and sex as the patients. These children had no cardiac diseases according to history and echocardiography.

### 2.3. Method

Echocardiography was performed with a GE Vivid 3 echocardiographic machine (GE Vingmed, Horten, Norway) using a 3-MHz probe with pulsed Doppler tissue imaging software. All M- mode, two-dimensional, Doppler, and pulse tissue Doppler echocardiographic measures were performed by one qualified cardiologist. Moreover, ejection fraction, shortening fraction, and septal and posterior wall thickness in systole and diastole were measured in the left parasternal long-axis view. In addition, the aortic annulus was measured in the parasternal long-axis view at the hinge points of the valves, aortic sinus, and the site of the connection of sinus to ascending aorta.

The pulsed Doppler sample volume was placed at the mitral valve and tricuspid tips and three cardiac cycles were recorded from the apical window. Early (E) and late (A) peak velocities and their ratio were determined for evaluation of diastolic function.

Pulse Doppler tissue imaging was obtained with the sample volume placed at the lateral corner of the mitral annulus and subsequently on the medial (or septal) and tricuspid corner in the apical four-chamber view. In each region, systolic (S) wave and early (Ea) as well as late (Aa) diastolic velocities were recorded.

### 2.4. Preoperative Clinical Data

Preoperative data assessment included age, sex, age at operation, present age, body weight, hemoglobin content, pre-operational echocardiographic findings, and any previous cardiac operation or interventional treatment. It should be noted that no pre-operational angiography was done, except for one patient with pre-operational atrial septostomy.

### 2.5. Arterial Switch Operation Description in short

ASO was performed under systemic anesthesia using low-flow (i.e., 50-100 mL/kg/min) hypothermic (range, 28° to 32°C) cardiopulmonary bypass. After cannulation and starting the cardiopulmonary bypass, ductus arteriosus was ligated, division of aorta and pulmonary artery was done, and after transfer of the coronary arteries, reconstruction of the pulmonary artery was performed with autologous pericardium in all the patients. In the patients with ventricular septal defect, Gortex patch was used to repair the ventricular septal defect.

### 2.6. Postoperative Management

Arterial blood pressure and central venous pressure were invasively monitored and surface as well as core temperatures were measured. No left atrial pressure monitoring was made. For sedation, fentanyl and midazolam were continuously administered. Inotropic drugs, such as intravenous injections of moderate-to-low doses of dopamine, nitroglycerin, or epinephrine, were used, as well. Pressure support mechanical ventilation was also applied.

### 2.7. Palliative Operations

Three patients (1 patient with simple TGA for left ventricular training, 2 patients with TGA and ventricular septal defect) underwent PA banding operation. ASO was performed in these patients when they were ready for operation.

### 2.8.Statistical Analysis

The statistical analyses were performed using the SPSS statistical software, version 16 (SPSS, Inc. Chicago, IL). The data were expressed as mean ± one standard deviation. Student’s t-test was used to compare the mean values of the patients and the control group with the probability values being statistically significant at the 0.05 levels. Besides, Pearson correlation was used to evaluate the relationship between the parameters.

## 3. Results

The mean follow up time was 40.9±5.6 months. There were 20 males and 13 females in both the study group and the control group. The intra-operative and pre-operative results are shown in Table 1. The median age at operation was 23 days.

**Table1.** Operative and Postoperative Clinical Data in the Patients Group

Variable	mean±SD
Operation time (hour)	5.15±0.72
Cardiopulmonary bypass time (minute)	126.98±31.83
Aortic cross clamp time (minutes)	86.48±13.51
Intensive care admission times (hour)	105±32
Need for mechanical ventilation (hour)	62.5±87.58
Total admission time (day)	8.1±4.82
Need for re-intubation	3 (n)

### 3.1. Need for Inotrope and Antiarrhythmic

The most commonly used inotrope was dopamine which was used in all the patients after the operation. Epinephrine and milrinone were also used in 18 and 25 patients, respectively. On the other hand, the less commonly used drugs were norepinephrine (2 patients) and phenylephrine (1 patient). Besides, only in 3 cases, amiodarone was used as an antiarrhythmic drug postoperatively in order to control the junctional rhythm (2 patients) and ventricular tachycardia (1 patient).

### 3.2. Relevant Anatomy at Operation

The relative anatomic position between the aorta and the pulmonary artery was anteroposterior in 21% (7 of 33 patients), right anterior and left anterior in 74% (24 of 33 patients), and right-left juxtaposition in 6% (2 of 33 patients) of the children. According to the Leiden classification for coronary artery anatomy in TGA and considering the data records presented in the operation sheets, 25 patients (75%) had normal [aLCXbR] coronary pattern. Other coronary patterns included aRLbCX in two (6%), aLbCXR in 4 (12%), bLCXR in one (3%), and bRLCX in one patient (3%).

In pre-operation evaluation, the atrial septal defect was reported in 10 patients (30.3%), patent ductus arteriosus in 31 patients (93.9%), mild tricuspid insufficiency in 8 patients (24.3%), mild pulmonary stenosis in 4 patients, and ventricular septal defect in 13 patients. No one had multiple ventricular septal defects.

### 3.3.Post Operation Echocardiographic Findings

#### 3.3.1. Valvular Problems

**Figure 1.**
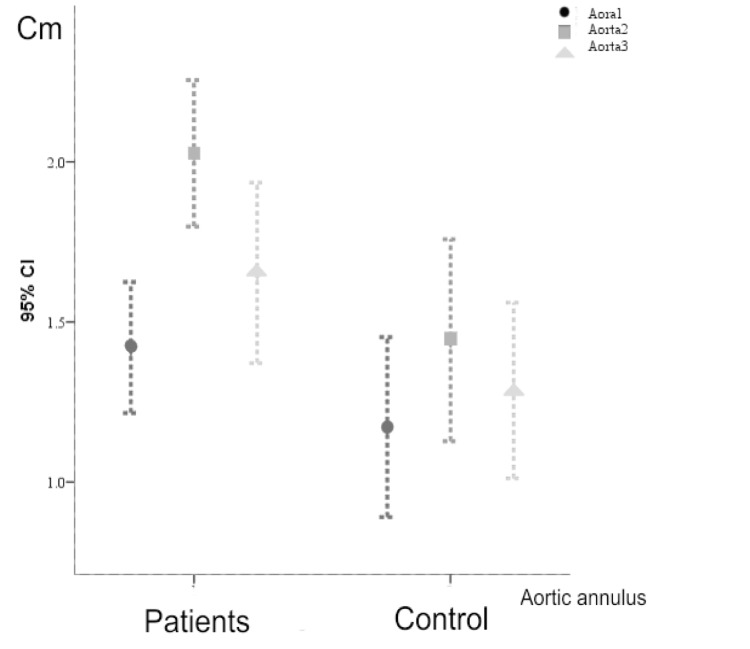
Error Bars Graph of Mean Aortic Annulus Measurements at: (Aorta 1) True Annulus, (Aorta 2) Sinus, and (Aorta 3) Sinotubular Junction of the Patients (2) and the Control Group (1)

Among the 33 patients with ASO, two patients had mild pulmonary stenosis (6%), ten patients had mild pulmonary insufficiency (30%), and one patient had moderate pulmonary insufficiency (3%). Aortic stenosis of trivial to mild degree was seen in 4 patients (12%). In addition, four patients had mild aortic insufficiency (12%) and one patient had moderate aortic insufficiency. Besides, no patient had mitral regurgitation and only one patient had mild tricuspid regurgitation. Pulmonary annulus diameter was significantly dilated in the patients in comparison to the controls (1.67±0.41 vs. 1.29±0.28, P≤0.001). Moreover, aortic annulus diameter (1.56±0.42 vs. 1.24±0.21, P=0.001) and aortic sinus diameter (2.06±0.41 vs. 1.44±0.34, P=0.002) were significantly dilated, while the sinutuboar junction diameter (1.65±0.5 vs. 1.28±0.29, P=0.094) was not dilated (Figure 1).

#### 3.3.2. M-mode Echocardiography

No statistically significant difference was observed between the patients and the controls regarding M-mode echocardiographic parameters (Table 2).

**Table 2.** Comparison of the Patients and Controls Regarding M-Mode Echocardiographic Parameters

M -mode	Patients[Table-fn fn5532](mean±SD)	Control (mean±SD)	P value
IVSd (Cm)	0.66±0.18	0.67±0.19	0.770
IVSS (Cm)	0.89±0.25	0.84±0.2	0.421
LVIDd (Cm)	3.01±0.84	3.15±0.54	0.464
LVIDs (Cm)	1.52±0.53	1.77±0.32	0.034
LVPWd (Cm)	0.56±0.12	0.52±0.15	0.210
LVPWS (Cm)	0.72±0.15	0.68±0.19	0.377
EDV (Cm3)	32.46±17.3	40.43±16.2	0.071
ESV (Cm3)	8.61±4.97	9.86±4.56	0.338
EF (%)	73.78±8.67	75.51±5.72	0.352
SV (Cm3/s)	24.96±12	30.40±12.5	0.100
FS (%)	41.93±8	43.42±5.62	0.401

^Abbreviations:^LVSID, Left ventricular systolic diameter; LVEDd, Left ventricular end diastolic diameter; LVESV, Left ventricular end systolic volume; LVPWS, Left ventricular posterior wall thickness at systole; LVPWd, Left ventricular posterior wall thickness at diastole; ESV, end systolic volume; FS ,Fractional shortening; EF ,ejection fraction

#### 3.3.3. Pulse Doppler Study

The results revealed a significant difference between the patients and the controls regarding the velocity of atrial contraction of tricuspid (AT), atrial velocity of mitral valve (AM), and ratio of early velocity to atrial velocity of mitral (EM/AM) (Table 3).

**Table 3.** Comparison of the Patients and Controls Regarding Pulse Doppler Echocardiographic Indexes

	Patients (mean±SD)	Control (mean±SD)	P value
E_T_ (cm/s)	0.72±0.26	0.66±0.14	0.301
A_T_ (cm/s)	0.68±0.19	0.48±0.16	0.000
E_M_ (cm/s)	1.01±0.23	1.05±0.17	0.422
A_M_ (cm/s)	0.68±0.28	0.57±0.13	0.041
E_T_[Table-fn fn5533]to A_T_	1.15±0.56	1.48±0.45	0.532
E_M_ to A_M_	2.52±5.4	1.90±0.47	0.011

Abbreviations: ET, Early velocity of tricuspid valve; AT, Atrial contraction velocity of tricuspid; EM, Early velocity of mitral valve; AM, Atrial contraction velocity of tricuspid

#### 3.3.4. Tissue Doppler Study

The patients and the controls were significantly different considering the lateral tricuspid annulus velocities in pulse tissue Doppler. The variables of tissue Doppler study are shown in Table 4.

**Table 4.** Comparison of the Patients and Controls Regarding Tissue Doppler Echocardiographic Indexes

	Patients group[Table-fn fn5534](mean±SD)	Control group (mean±SD)	P value
Tricuspid			
S (m/s)	0.08±0.01	0.12±0.01	<0.001
Ea (m/s)	0.12±0.03	0.150.02	<0.001
Aa (m/s)	0.09±0.03	0.12±0.03	< 0.001
Septum			
S (m/s)	0.05±0.02	0.07±0.01	< 0.001
Ea (m/s)	0.12±0.02	0.13±0.03	0.05
Aa (m/s)	0.08±0.02	0.08±0.02	0.55
Mitral			
S (m/s)	0.07±0.02	0.07±0.01	0.42
Ea (m/s)	0.13±0.03	0.15±0.03	0.14
Aa (m/s)	0.07±0.02	0.07±0.02	0.40

Abbreviations: S, Systolic velocity; Ea, early diastolic velocity; Aa, atrial contraction velocity

#### 3.3.5.Correlation between the Factors

Bivariate analysis of the variables within the patients’ group showed correlations between several variables. Aortic cross clamp time was statistically correlated to the age at operation (r=0.699 and P<0.001). However, no statistically significant correlation was found between the cross clamp time and the need for mechanical ventilation or hospital admission. Considering the use of inotropes, the duration of using dopamine after ASO was correlated to the cardiopulmonary bypass time (r=0.41, P=0.02) and aortic cross clamp time (r=0.45 and P=0.01). Nevertheless, the need for other inotropes had no relationships with other variables, including cardiopulmonary bypass time and aortic cross clamp time. The use of milrinone pre-operatively showed correlations with the present echocardiographic findings of the left ventricular systolic diameter (r=0.44, P=0.03), left ventricular posterior wall diameter (r=0.40, P=0.03), and systolic tissue Doppler velocities of lateral tricuspid annulus (r=0.45, P=0.03). Relationships were also found between the pump time and stroke volume (r=-0.58, P=0.004), intervetricular diameter in systole (r=-0.686, P=0.001), intervetricular diameter in diastole (r=-0.574, P=0.001), and left ventricular systolic diameter (r=-0.611, P=0.001). Moreover, the duration of cross clamp time was negatively related to ejection fraction (r=-0.387, P=0.043), intervetricular diameter in systole (r=-0.68, P=0.001), intervetricular diameter in diastole (r=-0.59, P=0.001), and left ventricular diastolic diameter (r=-0.66, P=0.001).

## 4. Discussion

This study reported the mid-term echocardiographic outcomes of the patients with TGA who underwent ASO. In our study, echocardiographic parameters of cardiac function as well as the function and diameter of neoaortic and neopulmonic valves were evaluated. We also assessed the correlation between these parameters and some perioperative variables, including age at operation, cardiopulmonary bypass time, aortic cross clamp time, need for inotropes, accompanied anatomical malformations, hospital admission time, need for mechanical ventilation, and hemodynamic parameters, within the first 12 hours postoperatively. Most patients were neonates with a median age of 23 days. All the patients, except for three, had a single stage ASO and almost 39% had complex TGA. The incidence of unusual coronary patterns was similar to that seen in the literature, with 65% of the patients having type A and type B, 18% having type C, and 6% having a single coronary artery according to Yacub classification (7).

When ASO for TGA was introduced in the early to mid-1980s, surgical mortality was high.

During the past 25 years, the results of ASO have improved substantially. The mortality rate has decreased from approximately 20% in the early 1980s to 3% in the full-term infants with TGA who are operated in the first 2 weeks of life (8).

In addition to well-recognized complications, such as pulmonary artery stenosis and coronary artery obstruction, several studies have reported the dilatation of the aortic root in childhood. Due to these findings, one of the major concerns of ASO nowadays is the possible deterioration of neoaortic valve function during growth and long-term follow-up (9).

ASO is the procedure of choice in treatment of TGA although the assessment of late mortality and morbidity predictors with special regards to the neoaortic valve function, the reconstructed pulmonary artery, and the fate of the implanted coronary arteries is required prior to any judgment. Few reports have analyzed these parameters within the same series (2). In our study with a mean follow up of 40 months after the operation, the prevalence of aortic regurgitation was 12 % which is in accordance with other publications (10-12).

Occurrence of aortic regurgitation was observed early in the ASO experience and reported in 30% and 55% of the patients for whom a two-stage operation was performed. Later, when primary repair or rapid two-stage operation was the rule, the prevalence of aortic regurgitation decreased and ranged between 5% and 22% after a one- to two-year follow-up (13, 14). In most of the recent publications with a longer follow-up (around five years), aortic regurgitation is a rare complication with a prevalence between 0.3% and 10% (12). One exception was a study reporting a prevalence of 30% after 5.8 years of follow-up (10). Other investigators found that some predictive preoperative risk factors of development of aortic regurgitation were the aorta and pulmonary artery size discrepancy, presence of aortic regurgitation at discharge, pulmonary artery banding, complex transposition of great artery, aortic arch obstruction, and older age at ASO (11, 13). In our study, no difference was found between the patients who had two-stage and one-stage operations; however, the patient with TGA and ventricular septal defect had more aortic regurgitation compared to those with simple TGA.

Although neoaortic insufficiency does not represent a matter of concern in the majority of the patients, careful echocardiographic follow-up is mandatory. Progression of neo-aortic valve regurgitation after ASO has been reported in childhood (11). Moreover, a study showed that aortic root dilation could occur after ASO, but it was not progressive later in childhood (13).

Neoaortic annulus and root were measured at true annular, sinus, and sinotubular junction and were larger than normal at all the three points. We found trivial to mild AS at valvular or supravalvular areas in 12% of the patients without any echocardiographic or clinical explanation. Left ventricular outflow tract and neoaortic stenosis are very rare in the literature (15). In a multi-institutional study, Williams et al. (16) reported that six out of 514 neonates with TGA had neoaortic stenosis. Also, most of the measured gradients were not hemodynamically significant. Only 6% of our patients had mild pulmonary stenosis (gradient=25-40mmhg). The patients had no preoperational aortic stenosis as the post operation source of neopulmonary stenosis.

In one study, the most common complication in the survivors was stenosis of the pulmonary arteries (supravalvular and branch pulmonary artery stenosis) with a reported incidence of 7% to 40% (17). Right ventricular outflow tract obstruction represents the most common reason for reoperation after ASO. Overall, several authors have postulated that pulmonary reconstruction using a single “pantaloon-shaped” pericardial patch results in normal pulmonary artery growth and lower right ventricular outflow tract pressure gradients with a decreased incidence for reoperation (18). In this study, we also used the pericardial patch in the same manner of pantaloons shape. However, several studies have failed to show any superiority of the three different surgical techniques. Low right ventricular outflow tract gradients have also been observed by Carrell et al. using the direct pulmonary anastomosis (18, 19). However, because each suture line is associated with the development of fibrous tissue, even in the presence of reabsorbable sutures, one may speculate that in the occurrence of unimpaired growth of the main pulmonary artery, discrete circumferential narrowing will result from the former suture line that will be detected by sensitive Doppler echocardiography. In most of the studies presented in the literature, mild transpulmonary gradients were observed in most of the patients, independent of the employed surgical technique. In contrast to the technique of direct pulmonary anastomosis, pulmonary reconstruction using pericardial patch material did not show any change in the pressure gradients over time, indicating sufficient enlargement of the coronary explanations sites either with one “pantaloon shaped” pericardial patch or two free pericardial patches.

The prevalence of mild and moderate postoperative pulmonary regurgitation in our patients was 30% and 3% (one patient), respectively. The patient with moderate pulmonary regurgitation was a 5-year-old boy, a case of very late operation (1 year old), with pre operation pulmonary artery- banding and moderate aortic regurgitation who is now on anti failure therapy. Overall, pulmonary regurgitation after ASO seems to have been neglected in all the previous studies. However, neopulmonary valve regurgitation has been reported to occur after ASO, with most studies using Doppler echocardiographic evaluation and the reported incidence varying from 9% to 80% (15). The patients with pre-operative mild left ventricular obstruction had no post operative gradient. Other studies also showed that the preoperative dynamic left ventricular outflow tract obstruction disappeared after the ASO (15).

The results of the current study showed that at the baseline, midterm left ventricular contractility was normal in all the patients with TGA after ASO. Older age at operation, longer cardiopulmonary bypass time, longer circulatory arrest time, and unusual coronary artery pattern were not associated with impaired left ventricular contractility. Moreover, ventricular function and contractility were found to have no significant relationships with the period of time since surgery. However, the duration of milrinone use post operatively had a correlation with left ventricular systolic diameter, left ventricular posterior wall diameter, and tissue Doppler indexes of lateral tricuspid annulus.

Left ventricular function is usually normal after the ASO. A comparative study between the arterial and atrial switch showed that late postoperatively, left ventricular ejection fraction was within the normal range in 98% of the patients with simple TGA undergoing the arterial switch repair, but in 79% of those who underwent an atrial switch repair (15). Good left ventricular systolic function was reported in older children (17) and severe left ventricular systolic dysfunction is not expected in the absence of coronary artery abnormalities. Other investigators have reported normal left ventricular mass, volume, ejection fraction, dimension, shortening fraction, stress- velocity index, and stress-shortening index in the patients who have previously undergone an ASO for TGA with either intact ventricular septum or ventricular septal defect. Although some investigators have reported depressed function after ASO, they have included the patients who have undergone a prolonged period of preparatory pulmonary artery banding, making the data more difficult to interpret. Furthermore, although the elevated end-diastolic volume has been occasionally observed after ASO, a large percentage of these patients had residual aortic regurgitation and residual ventricular septal defect that seem to be the cause of left ventricular dysfunction (17).

Left ventricular dysfunction has been suggested to be related to ischemic damage caused by coronary insufficiency or preoperative ischemia or later by coronary kinking or valvular insufficiency. A previous study (20), however, demonstrated no areas of myocardial infarction in the patients after the ASO without ischemic events. Similarly, no previous ischemic events were reported in our patients’ group and myocardial scar and coronary imaging were not included in the imaging protocol.

The patients’ E and A and E/A showed impaired right ventricular relaxation compared to the control patients. Comparison of the regional myocardial pulse tissue Doppler in the patients also showed a significantly decreased velocity of the lateral tricuspid annulus compared to the control group. Overall, the parameters derived from tissue Doppler echocardiography need further validation in comparison to other invasive and non-invasive methods.

## 5. Conclusion

This study revealed good and preserved left ventricular function in the patients, but some abnormalities in lateral tricuspid tissue Doppler velocities. Neoaortic and pulmonary diameters were significantly dilated, while aortic and pulmonary insufficiencies were clinically insignificant in most of the patients. Long-term follow-up is necessary in these patients.
